# Generation of clinical-grade human induced pluripotent stem cells in Xeno-free conditions

**DOI:** 10.1186/s13287-015-0206-y

**Published:** 2015-11-12

**Authors:** Juan Wang, Jie Hao, Donghui Bai, Qi Gu, Weifang Han, Lei Wang, Yuanqing Tan, Xia Li, Ke Xue, Pencheng Han, Zhengxin Liu, Yundan Jia, Jun Wu, Lei Liu, Liu Wang, Wei Li, Zhonghua Liu, Qi Zhou

**Affiliations:** State Key of Stem Cells and Laboratory of Reproductive Biology, Institute of Zoology, Chinese Academy of Sciences, Beijing, 100101 China; College of Life Science, Northeast Agricultural University of China, Harbin, 150030 China; Graduate School of Chinese Academy of Sciences, Beijing, 100049 China

## Abstract

**Introduction:**

Human induced pluripotent stem cells (hiPSCs) are considered as one of the most promising seed cell sources in regenerative medicine. Now hiPSC-based clinical trials are underway. To ensure clinical safety, cells used in clinical trials or therapies should be generated under GMP conditions, and with Xeno-free culture media to avoid possible side effects like immune rejection that induced by the Xeno reagents. However, up to now there are no reports for hiPSC lines developed completely under GMP conditions using Xeno-free reagents.

**Methods:**

Clinical-grade human foreskin fibroblast (HFF) cells used as feeder cells and parental cells of the clinical-grade hiPSCs were isolated from human foreskin tissues and cultured in Xeno-free media. Clinical-grade hiPSCs were derived by integration-free Sendai virus-based reprogramming kit in Xeno-free pluriton™ reprogramming medium or X medium. Neural cells and cardiomyocytes differentiation were conducted following a series of spatial and temporal specific signals induction according to the corresponding lineage development signals. Biological safety evaluation of the clinical-grade HFF cells and hiPSCs were conducted following the guidance of the “Pharmacopoeia of the People's Republic of China, Edition 2010, Volume III”.

**Results:**

We have successfully derived several integration-free clinical-grade hiPSC lines under GMP-controlled conditions and with Xeno-free reagents culture media in line with the current guidance of international and national evaluation criteria. As for the source of hiPSCs and feeder cells, biological safety evaluation of the HFF cells have been strictly reviewed by the National Institutes for Food and Drug Control (NIFDC). The hiPSC lines are pluripotent and have passed the safety evaluation. Moreover, one of the randomly selected hiPSC lines was capable of differentiating into functional neural cells and cardiomyocytes in Xeno-free culture media.

**Conclusion:**

The clinical-grade hiPSC lines therefore could be valuable sources for future hiPSC-based clinical trials or therapies and for drug screening.

**Electronic supplementary material:**

The online version of this article (doi:10.1186/s13287-015-0206-y) contains supplementary material, which is available to authorized users.

## Introduction

Human pluripotent stem cells (hPSCs) can differentiate into any type of cells in the body, such as functional neural progenitor cells or cardiomyocytes, and therefore have enormous value in regenerative medicine. The increasing incidence of degenerative diseases, limitations of traditional therapeutic methods, and the shortage of isolated human functional cells have urged scientists to turn to stem cell-based cell replacement therapies. Although the translation from basic discoveries to clinical settings comes with great challenges, intensive stem cell-based clinical trials are emerging from around the world. For human embryonic stem cells (hESCs), a clinical trial of spinal-cord injury treatment using immature glial cells derived from hESCs by the Geron Corporation (Menlo Park, California, USA) has recommenced after it was brought to a halt in 2011 [1]. Another clinical trial of hESCs involving the generation of retinal pigmented epithelial (RPE) cells for the treatment of eye disorders such as Stargardt’s macular dystrophy, myopic macular degeneration, and advanced dry age-related macular degeneration is currently being conducted by the Advanced Cell Technology company (Marlborough, Massachusetts, USA) in America [2]. The mid-term outcomes confirmed the safety and efficacy of hESC-derived RPE in patients [3]. When taking moral and ethical aspects into consideration, human induced pluripotent stem cells (hiPSCs) are more ideal and feasible cell sources for transplantation compared with hESCs. A clinical trial for eye disorder treatment using hiPSC-derived RPE cells is also now being carried out in Japan [4].

Initially, the generation of hiPSCs involved integrated retrovirus expressing *Oct4*, *Sox2*, *Klf4*, and *c-Myc* [5, 6]. However, random integrations may result in insertional mutagenesis consequently risking patients’ safety. Also, unexpected activation of the integrated oncogene *c-Myc* may initiate tumorigenesis [7]. To circumvent the aforementioned problems, integration-free hiPSCs have been generated using Sendai viruses [8], episomal vectors [9], mRNAs [10], minicircle DNAs [11], microRNAs [12], and proteins [13]. Although each method has its own merits and disadvantages, integration-free reprogramming methods are optimal for future clinical applications.

Most of the hESC lines collected by the National Institutes of Health (NIH) have been reported ineligible for future therapeutic products use because their derivation processes did not follow the “Tissue Donor Guidance” [14]. Precautionary actions are therefore of utmost importance in order to ensure the safety, effectiveness, traceability, reproducibility, and legality of hiPSCs intended for clinical trials or therapies. Careful screening for legal and eligible donors is a very important step. According to the current national and international regulation policies, most countries require a good manufacturing practice (GMP) environment when handling the cells [15, 16]. Reagents used in the culture process will greatly affect the safety and quality of the cells. Xeno reagents would not only increase the risk of infections but also cause immune rejection upon cell transplantation [17]. Almost all countries have advocated that animal reagents should not be used in cells for clinical applications [18]. Therefore it is sensible to use Xeno-free reagents in all cell handling processes. To further ensure the safety of the cells used in clinical settings, endotoxin and serious pathogenic microorganism such as mycoplasma and HIV virus have to be tested [19].

We define hiPSCs intended to be used for potential clinical applications as clinical-grade hiPSCs. Theoretically, clinical-grade hiPSCs should meet the following requirements. First, parental cell donors should meet the requirements of the “Tissue Donor Guidance”. Second, the cell handling processes should be conducted under GMP-controlled environments and with Xeno-free reagents. Third, the derived clinical-grade hiPSCs should be integration-free and biologically safe.

Previously, a group reported the derivation of hiPSCs under fully defined conditions. However, the biological safety of the obtained hiPSC lines is questionable. Furthermore, the group did not describe whether their protocol was within the “Tissue Donor Guidance” [20]. Several other groups have also successfully achieved the so-called clinical-grade hiPSC lines by converting the existing nonclinical-grade hiPSC lines. Still, in these processes animal products were applied in the initial stages, which may confer risks of cross-species infectious agent transfer [21–23]. For these reasons, there is an urgent need to derive clinical-grade hiPSC lines strictly following all requirements of clinical-grade hiPSCs.

Here, we report successful generation of several clinical-grade hiPSC lines. A series of pathogenic microorganism tests were conducted on foreskin tissue donors according to the “Tissue Donor Guidance” of China. The human foreskin fibroblast (HFF) cells used for reprogramming and as feeder cells were isolated and cultured using Xeno-free reagents in a GMP-grade laboratory and were confirmed negative for mycoplasma and certain pathogenic microorganisms. Furthermore, the biological safety of the HFF cells is validated by the National Institutes for Food and Drug Control (NIFDC). All processes of the hiPSC generation were conducted under GMP-controlled conditions using Xeno-free media and reagents. The resulting hiPSCs are validated as integration-free, and are able to randomly differentiate into three germ layers in vitro and in vivo as well as directly differentiate into functional neural cells and cardiomyocytes in Xeno-free media. The biological safety of these hiPSCs is further validated. These hiPSC lines, generated following all of the requirements of clinical-grade hiPSCs strictly, might serve as valuable cell sources for future clinical applications.

## Methods

### Ethical approval

The foreskin tissues were donated by males undergoing foreskin operation in Beijing Children’s Hospital with the approval of the Animal and Medical Ethics Committee of Institute of Zoology, Chinese Academy of Sciences. Written consent was obtained from the donors’ parents. The content of the consent includes the potential use of cells derived from the foreskin (research or potential clinical therapies) and the rights and obligations of the donors. After donation, a thorough assessment of the donors’ medical history and infectious diseases were performed. Based on the results, donors were selected. All information is secured to protect the privacy and confidentiality of donors. No financial benefits were involved in the donation process. All cell isolation and culture procedures followed closely the guidelines legislated and posted by the Ministry of Health of the People’s Republic of China.

All animal experiments were approved in advance by the Animal and Medical Ethics Committee of Institute of Zoology, Chinese Academy of Sciences.

### Isolation of fibroblasts from foreskin tissues

Foreskin tissues were collected into 50 ml centrifuge tubes containing Dulbecco's Phosphate Buffered Saline (DPBS)-CTS (Life Technologies, Carlsbad, California, USA) and transported to the laboratory on ice. After sufficient washing with DPBS-CTS, fat and connective tissues were removed from the foreskin. The foreskin was then cut into small pieces with a volume of about 1 mm^3^ and transferred to T75 flasks precoated with MesenCult-XF attachment substrates (Stem Cell Technologies, Vancouver, British Columbia, Canada) without cell culture medium. After 24 hours of inverted culturing in a humidified incubator (at 37 °C, 5 % CO_2_), 4 ml FibroGRO™ Xeno-Free Human Fibroblast Expansion Medium (Millipore, Billerica, Massachusetts, USA) was added to each flask. After 7 days of culture, fibroblasts could outgrow the tissues and be routinely passaged. The obtained fibroblasts were used as parental cells for hiPSC induction and as feeder cells after being irradiated with gamma rays.

### Cell culture

HFF cells were cultured in FibroGRO™ Xeno-Free Human Fibroblast Expansion Medium (Millipore) on dishes precoated with Mesencult-XF attachment substrates according to the manufacturer’s instructions. Clinical-grade hiPSCs were initially cultured on gamma ray-inactivated human feeder cells in pluriton™ reprogramming medium (Stemgent, Lexington, Massachusetts, USA) or X medium (configured in our laboratory). The formulations of X medium were as follows: clinical-grade hESC basic medium and clinical-grade N2B27 medium mixed at 1:1 ratio supplemented with 10 ng/ml human leukemia inhibitory factor (hLif; Peprotech) and human basic fibroblast growth factor (bFGF; Peprotech, Rocky Hill, Rocky Hill, USA). The clinical-grade hESC basic medium contained knockout Dulbecco’s modified Eagle’s medium (DMEM)-CTS (Life Technologies), 20 % knockout serum replacement (KOSR)-CTS (Life Technologies), 100 μM none essential amino acid (NEAA; Life Technologies), 2 mM GlutaMAX-CTS (Life Technologies), 55 μM β-mercaptoethanol (Life Technologies), and 1000 U/ml penicillin/streptomycin (Life Technologies). The clinical-grade N2B27 medium was a mixture of knockout DMEM-CTS supplemented with N2-CTS (Life Technologies) and Neurobasal Medium-CTS (Life Technologies) supplemented with B27-CTS (Life Technologies) at 1:1 ratio plus 100 μM NEAA, 2 mM GlutaMAX-CTS, 55 μM β-mercaptoethanol, and 1000 U/ml penicillin/streptomycin. At passages 10–12, the cells were transferred to E8 medium (Life Technologies) without feeder cells.

### Induction of hiPSCs from HFF cells

HFF cells were cultured in six-well sterile culture plates precoated with MesenCult-XF Attachment Substrate in FibroGRO™ Xeno-Free Human Fibroblast Expansion Medium without antibodies. The CytoTune®-iPS Sendai Reprogramming Kit (Life Technologies) was used for deriving integration-free iPSCs according to the manufacturer’s instructions. The medium used in the reprogramming process was pluriton™ reprogramming medium or X medium. Colonies with typical hPSC morphology were picked 20 days post transduction for further expansion.

### Alkaline phosphatase staining

Alkaline phosphatase staining was performed using the Alkaline Phosphatase Assay Kit (Beyotime, Shanghai, China) according to the manufacturer’s instructions.

### Immunofluorescence staining

Immunofluorescence staining was conducted as described previously [23]. The primary antibodies used were OCT4 (1:200; Santa, Dallas, Texas, USA), SOX2 (1:200; Millipore), SSEA-1 (1:200; Millipore), SSEA-3 (1:200; Millipore), SSEA-4 (1:200; Millipore), TH1 (1:400; Millipore), TUJ1 (1:1000; Covance, Princeton, New Jersey, USA), NKX2.5 (1:200; Abcam, Cambridge, UK), and CTNT (1:200; R&D, Minneapolis, Minnesota, USA). The secondary antibodies were donkey anti-mouse IgG (H + L) conjugated to Cy3 (1:200; Jackson, Lansing, Michigan, USA), donkey anti-mouse IgG conjugated to Cy5 (1:200; Jackson), donkey anti-goat IgG conjugated to Cy5 (1:200; Jackson), donkey anti-rabbit IgG conjugated to fluorescein isothiocyanate (FITC) (1:200; Jackson), and donkey anti-rat IgG conjugated to Cy5 (1:200; Jackson).

### Karyotyping and G-binding

When hiPSCs reached 60–70 % confluency, karytoyping and G-binding analysis were carried out by Chinese Academy of Medical Science & Peking Union Medical College.

### RNA isolation, reverse transcription and PCR analysis

RNA isolation and reverse transcription were performed as described previously [24]. Real-time PCRs were conducted with the SYBR® Premix EX Taq™ kit (Takara, Minamikusatsu Station, Japan) and Agilent, Santa Clara, California, USA, Mx3005P equipment according to the manufacturer’s instructions. Normal PCR reactions were conducted according to the previous report [24]. Primer sequences used in the experiments are presented in Additional file 1.

### Embryoid body formation assay

The embryoid body (EB) formation assay was conducted as described previously [24] with the exception of using Xeno-free ethylenediamine tetraacetic acid (EDTA; Life Technologies) for cell dissociation and clinical-grade hESC basic medium for EB culture.

### Teratoma formation assay

Confluent cells in a six-well plate were harvested using EDTA and suspended in DPBS-CTS. Then 10^6^ cells in 20–30 μl DPBS were injected into each testis of severe combined immunodeficiency (SCID) mouse under a sterile stereo microscope. The mice were sacrificed 8 weeks after injection for teratoma examination following the guidelines of the Institutional Animal Care and Use Committee (IACUC). The teratomas were fixed, sliced, and stained with hematoxylin and eosin for further analysis.

### Neural cell differentiation

Neural cell differentiation was performed as described previously [25] apart from the media used being Xeno-free in all processes. Briefly, confluent hiPSCs were dissociated into single cells by tryple-CTS (Life Technologies) and plated on Vitronectin-coated dishes at a density of 4 × 10^5^ cells/cm^2^ on day 0. Initially, the cells were cultured in a medium of knockout DMEM-CTS supplemented with 15 % KOSR-CTS, 100 μM NEAA, 2 mM GlutaMAX-CTS, 55 μM β-mercaptoethanol, and 1000 U/ml penicillin/streptomycin. On day 5, the medium containing KOSR-CTS was gradually transitioned to N2 medium-CTS as described previously [25]. On day 11, the medium was changed to Neurobasal medium-CTS supplemented with 2 % B27-CTS. To induce differentiation, growth factors and small molecules were added as follows: 10 μM SB431542 (Stemgent) from day 0 to 5, 100 nM LDN93189 (Stemgent) from day 0 to 11, 3 μM CHIR99021 from day 3 to 13, 100 ng/ml SHH C25II (R&D), 2 μM purmorphamine (Stemgent), and 100 ng/ml FGF8 (R&D) from day 1 to 7, and 20 ng/ml brain-derived neurotrophic factor (BDNF; R&D), 0.2 mM ascorbic acid (Sigma, Louis, Missouri, USA), 20 ng/ml glial cell line-derived neurotrophic factor (GDNF; R&D), 1 ng/ml transforming growth factor type β3 (TGFβ3; R&D), 0.5 mM dibutyryl cAMP (Sigma), and 10 μM (DAPT) N-[(3,5-Difluorophenyl)acetyl]-L-alanyl-2-phenyl]glycine-1,1-dimethylethyl ester (Tocris, Bristol, Bristol, UK) from day 11 to 23.

### Cardiomyocyte differentiation

Confluent hiPSCs were digested into single cells by accutase (Life Technologies) and seeded on vitronectin-coated dishes at a density of 1.25 × 10^5^ cells/cm^2^. Then the cells were grown in E8 medium for 2–3 days to reach 100 % confluency. To improve the efficiency of single cell survival, 10 μM Y27632 were added on the first day. Culture medium used in the differentiation process was RPMI 1640 (Life Technologies) with a combination of chemically defined components (modified in our laboratory and data not published) which we named Car-CDM. From day 0 (the day for differentiation medium adding) to day 8, Car-CDM without insulin was used as differentiation medium. In this period, the medium was changed on days 0, 1, 3, and 5. From day 0 to 1, 4 μM were added, whereas 2 μM Wnt-C59 (Sigma) was added from day 3 to 8 (Sigma). From day 8 onward, the differentiation medium was changed to Car-CDM plus insulin and the medium was changed every other day up to day 14. Beating cardiomycytes generally appeared on day 12 of differentiation.

### Endotoxin testing

Endotoxin testing was performed using the ToxinSensor™ Gel Clot Endotoxin Assay Kit (GeneScript, Piscataway, New Jersey, USA) according to the manufacturer’s instructions.

### Mycoplasma testing

Mycoplasma testing was performed using the PlasmoTest™ Reagent Kit (InvivoGen, San Diego, California, USA) according to the manufacturer’s instructions.

### Pathogenic microorganism testing

The defined pathogenic microorganisms tested were human papilloma virus (HPV), human parvovirus B19, human immunodeficiency virus (HIV), John Cunningham virus (JCV), Epstein–Barr virus (EBV), human hepatitis C virus (HCV), human hepatitis A virus (HAV), human cytomegalovirus (HCMV), human T-lymphotropic virus I (HTLV-I), human hepatitis B virus (HBV), bovine virus, and porcine virus. These viruses were detected using corresponding kits produced by Zhongshandaan Company, Guangzhou, Guangdong, China. Some of the viruses were reviewed by Beijing Ai Puyi Medical Testing Center, Beijing, China. The hiPSCs were also inoculated into the chorioallantoic membrane and yolk sac of chicks according to the methods described in the *Chinese Pharmacopoeia* (third edition).

## Results

### Preparations for generation of clinical-grade hiPSCs

**Fig 1:**
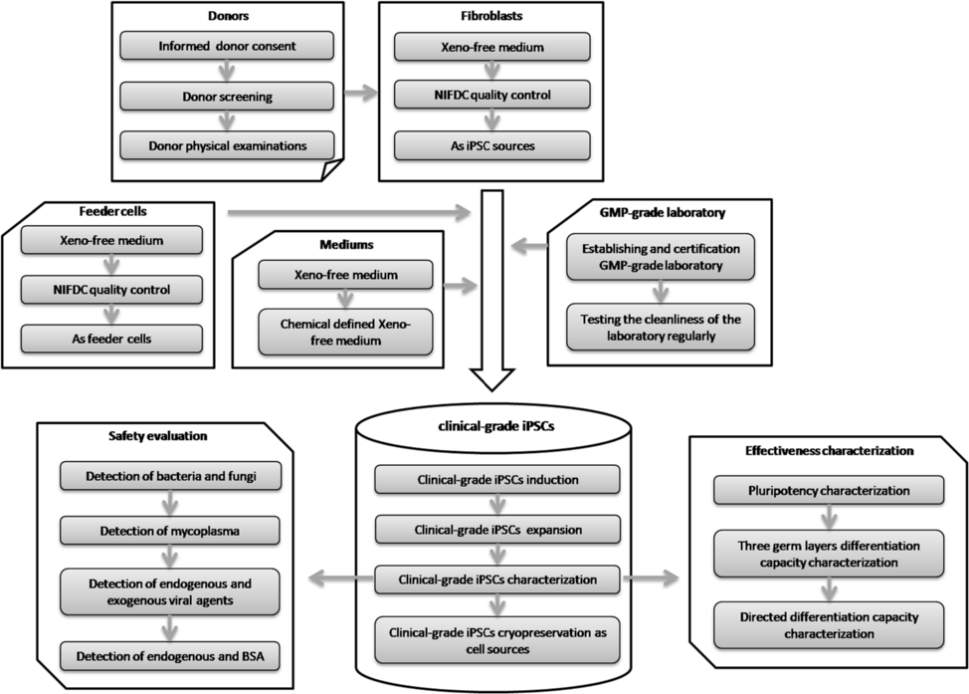
Overview of activities and requirements for clinical-grade hiPSC derivation and characterization. *GMP* good manufacturing practice, *iPSC* induced pluripotent stem cell, *NIFDC* National Institutes for Food and Drug Control, *BSA* Bovine serum albumin

Safety, traceability, and reproducibility are three basic criteria for cells to be used in clinical trials or therapies. To meet these criteria, GMP environments are required for the whole process of cell manipulation. We have established a GMP-controlled cell culture laboratory and it has passed the Beijing Institute for Drug Control (Beijing Center for Health Food and Cosmetics Control) test. The room was cleaned and tested regularly to ensure its cleanliness (Fig. 1).

The legal aspect is another fundamental requirement for cell-based therapy products. In China, the Guidelines for Human Somatic Cell Therapies and Quality Control of Cell-based Products stipulate that cell donation for clinical applications must comply with national requirements the same as for blood donation. In America, the Food and Drug Administration (FDA) “Tissue Donor Guidance (2007)” states that the potential tissue donor must test negative for a series of infectious microorganisms prior to donation. Screening for tissue donors who have signed the informed consent is therefore an important step to ensure the suitability of the cells generated from the donated tissue for clinical applications. After explaining the significance and potential applications of cells that would be generated from the donated tissue to children undergoing foreskin resection surgery and their parents, some patients and parents agreed to donate their tissues and signed the informed consent. The cases and medical reports of the patients were then strictly evaluated to assess their eligibility for donation. Finally, a 6-year-old boy’s foreskin tissue was chosen (Fig. 1).

### Derivation of clinical-grade HFF cells

Serum-containing medium are often used for fibroblast derivation and culture. To establish fibroblasts free of animal-source reagents in our laboratory, several types of Xeno-free or chemical defined human fibroblasts culture media were trialed (Data not shown). The result showed that FibroGRO™ Xeno-Free Human Fibroblast Expansion Medium provided the optimum support for HFF cell growth. As a potential clinical-grade hiPSC and Xeno-free feeder cell source, biosecurity of the HFF has to be validated. In this case, a series of biosecurity-related projects were carried out. The results of these projects are presented in Additional file 2. The cell properties and safety are validated by the NIFDC in China.

### Derivation of clinical-grade hiPSCs

**Fig. 2:**
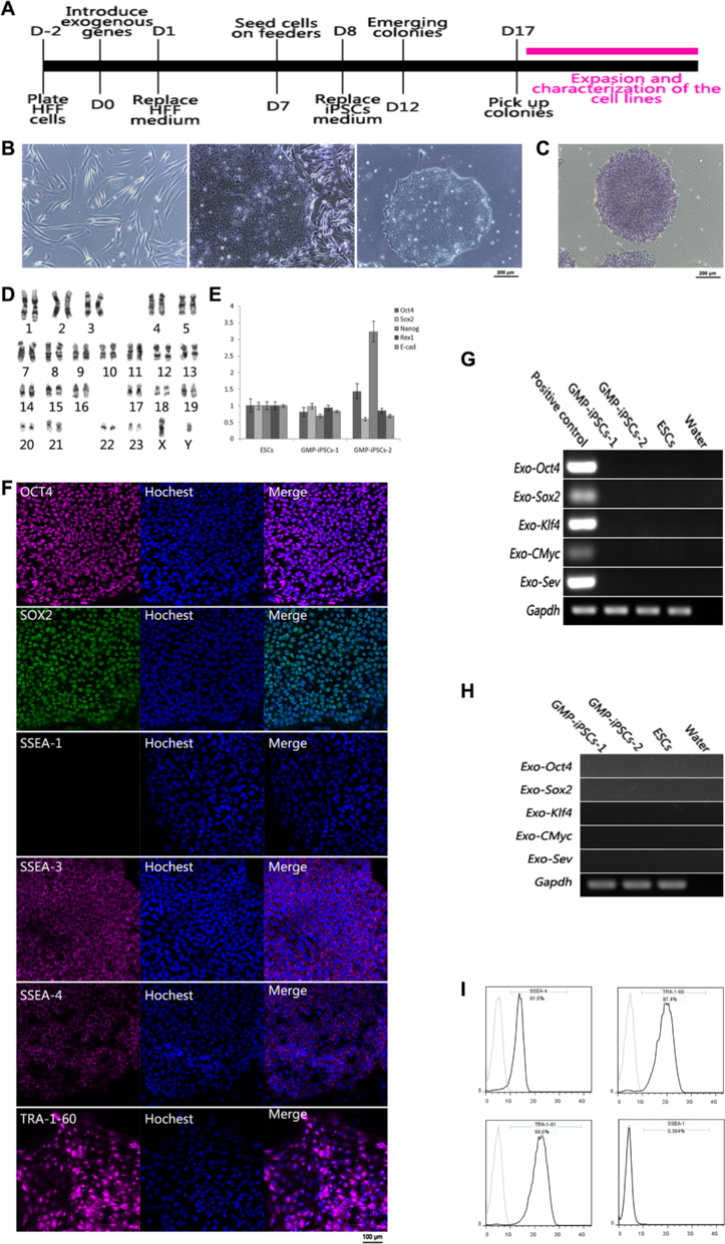
Reprogramming of clinical-grade HFF cells into clinical-grade hiPSCs and characterization of their pluripotency. **a** Time course of clinical-grade hiPSC generation. **b** Morphology of clinical-grade HFF cells, and clinical-grade hiPSCs grown on HFF feeder cells and in feeder-free conditions. Scale bar, 200 μm. **c** Clinical-grade hiPSCs express alkaline phosphatase. Scale bar, 200 μm. **d** Karyotyping results of one of the clinical-grade hiPSC lines. **e** mRNA expression levels of pluripotency-related genes of the clinical-grade hiPSCs relative to hESCs. **f** Immunofluorescence results of one of the clinical-grade hiPSC lines. The hiPSCs express pluripotency markers OCT4, SOX2, SSEA-3, SSEA-4, and TRA-1-60 at the protein level and do not express differentiation marker SSEA-1. Scale bar, 100 μm. **g** PCR analysis results show that the clinical-grade hiPSCs did not express Sendai virus exogenous genes. **h** PCR analysis results show that the Sendai virus genes did not integrate into the genomic of the clinical-grade hiPSCs. **i** Flow cytometry analysis of SSEA-1, SSEA-4, TRA-1-60, and TRA-1-81 expression of one of the clinical-grade hiPSC lines growing without feeders. *D* day, *ESC* embryonic stem cell, *GMP* good manufacturing practice, *HFF* human foreskin fibroblast, *iPSC* induced pluripotent stem cell, *SSEA* stage-specific embryonic antigen

The clinical-grade HFF cells of passage 8 were used for integration-free Sendai virus-based reprogramming. The reprogramming procedure was similar to the instructions of the CytoTune®-iPS Sendai Reprogramming Kit except that Xeno-free media were used in our protocol. Pluriton™ reprogramming medium is a type of Xeno-free medium which can efficiently support the generation of hiPSCs in mRNA-based reprogramming. The X medium, which was defined in our laboratory, could greatly improve the induction efficiency of pig and human iPSCs [24, 26] and could be configured with Xeno-free reagents. We proposed that clinical-grade hiPSCs could also be efficiently derived in pluriton™ reprogramming medium and X medium, and thus both media were utilized in the reprogramming process. The hiPSC colonies emerged at day 12 after infection and were picked up 5 days later to generate stable cell lines (Fig. 2a). The reprogramming efficiency of X medium was almost three times that of the pluriton™ reprogramming medium (data not shown).

### Clinical-grade hiPSCs express specific pluripotency markers

In total, 20 clinical-grade hiPSC lines were generated and two random lines were selected for further characterization. Both clinical-grade hiPSC lines exhibited morphology consistent with that of hESCs and could be subsequently passaged on clinical-grade HFF feeder layers (Fig. 2b; and Figure S1A in Additional file 3). These cells expressed high levels of alkaline phosphatase (Fig. 2c; and Figure S1B in Additional file 3) and maintained normal karyotypes when being characterized at passage 20 (Fig. 2d; and Figure S1C in Additional file 3). Real-time PCR results showed that the two cell lines expressed pluripotent genes *Oct4 (Pou5f1)*, *Sox2*, *Nanog*, *Rex1*, and *E-Cadherin* at similar levels to hESCs (Fig. 2e). Immunofluorescence results showed that the two cell lines expressed pluripotency markers OCT4, SOX2, SSEA-3, SSEA-4, and TRA-1-60 and did not express differentiation marker SSEA-1 (Fig. 2f; and Figure S1D in Additional file 3).

Chemically defined E8 medium and vitronectin-coated surfaces have been validated for the support of hiPSC culture. Therefore, to avoid hiPSC cell culture instability stemming from variable human sourced albumin batches, E8 medium and vitronectin-coated surfaces were used in the culture of these two lines. Both lines grew well without major differentiation in E8 medium (Fig. 2b, h; and Figure S1A in Additional file 3). The percentages of SSEA-4, TRA-1-60, and TRA-1-81 expression were higher than 90 %, whereas less than 1 % of SSEA-1 was expressed (Fig. 2i).

PCR results showed that both lines were integration-free (Fig. 2g) and all of the four Sendai virus vectors were gradually lost in the cytoplasm (Fig. 2h).

### Clinical-grade hiPSCs can differentiate into all three germ layers

**Fig 3:**
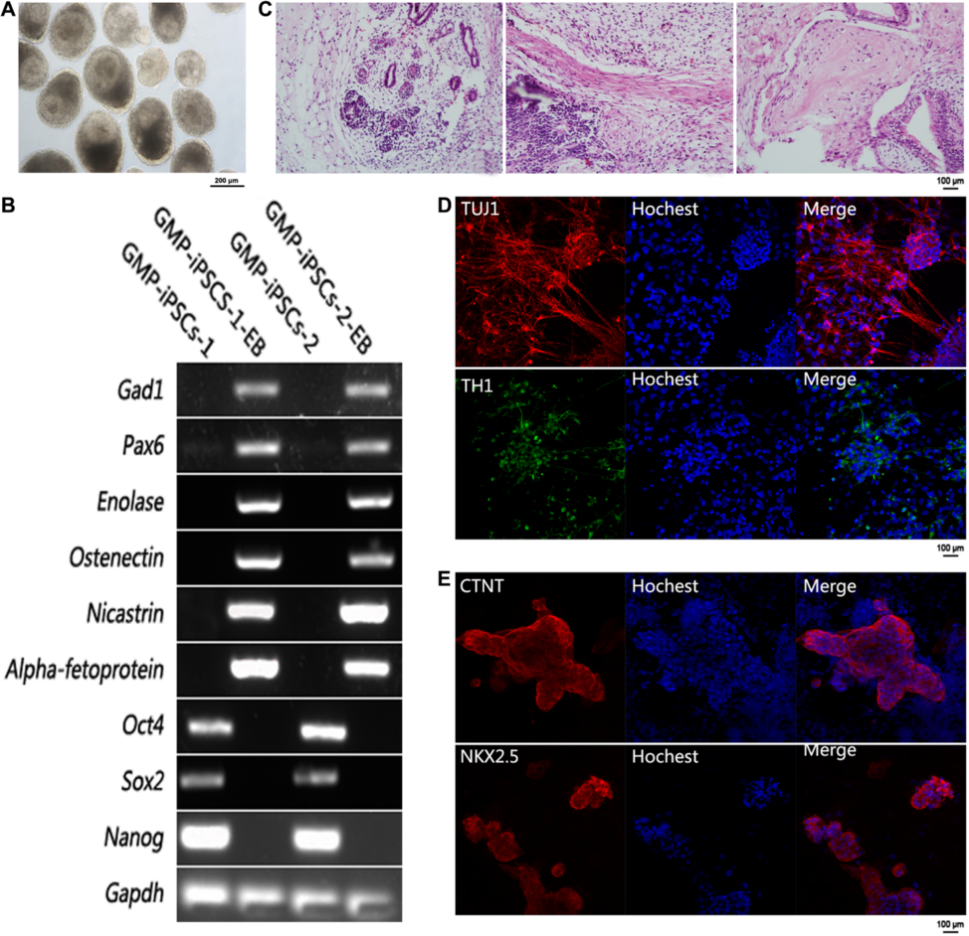
Clinical-grade hiPSCs can randomly differentiate into all three germ layers and directly differentiate into functional neural cells and cardiomyocytes in Xeno-free media. **a** Morphology of EBs at day 8 derived from one clinical-grade hiPSC line. Scale bar, 200 μm. **b** Gene expression profile for germ-layer marker genes of the day 8 EBs, ectoderm (*Gad1* and *Pax6*), mesoderm (*Enolase* and *Osteonectin*), endoderm (*Nicastrin* and *Alpha-fetoprotein*), and pluripotency (*Oct4*, *Sox2* and *Nanog*). **c** Hematoxylin and eosin staining of teratoma derived from one clinical-grade hiPSC line. Scale bar, 100 μm. The teratomas contain tissues of all three germ layers. **d** Immunofluorescence results of neural cells differentiated from one clinical-grade hiPSC line. The neural cells express TH1 and TUJ1 at protein level. Scale bar, 100 μm. **e** Immunofluorescence results of cardiomyocytes differentiated from one clinical-grade hiPSC line. The cardiomyocytes express NKX2.5 and CTNT at protein level. Scale bar, 100 μm. *EB* embryoid body, *GMP* good manufacturing practice, *iPSC* induced pluripotent stem cell

The EB and teratoma formation assays are commonly used to evaluate the differentiation ability of hPSCs. Both of the clinical-grade hiPSC lines were grown in suspension in clinical-grade hESC basic medium. Eight days later, they all formed round EBs (Fig. 3a; and Figure S1E in Additional file 3). RT-PCR analysis showed that the EBs expressed markers of all three germ layers, including ectoderm (*Gad1* and *Pax6*), mesoderm (*Enolase* and *Ostenectin*), and endoderm (*Nicastrin* and *Alpha-fetoprotein*) (Fig. 3b)*.* The teratoma formation assay was performed by injection of the two hiPSC lines to the testis of SCID mice separately. Two months later, teratomas were visualized in all injected testes. Histological analysis revealed that the teratomas comprised tissues of all three germ layers (Fig. 3c; and Figure S1F in Additional file 3).

### Clinical-grade hiPSCs can directly differentiate to functional neural cells and cardiomyocytes in Xeno-free media

hPSCs including hESCs and hiPSCs cannot be used directly in cell replacement therapies as they may induce tumor formation. However, terminally differentiated functional cells derived from hiPSCs can be used for clinical applications. Therefore it is important to evaluate the differentiation ability of our clinical-grade hiPSCs into specific cell types under Xeno-free conditions. Neural cell and cardiomyocyte differentiations were chosen for this purpose.

For neural cell differentiation, hiPSCs were directed to forebrain dopamine neurons following a series of spatial and temporal specific signal inductions according to the neural lineage development process [25]. Twenty-three days after differentiation, the neurons expressed neural marker TUJ1 and dopamine neural marker TH1 (Fig. 3d).

For cardiomyocyte differentiation, the Car-CDM medium modified in our laboratory was used. The differentiation process followed a temporal WNT signal activation and inhibition as described previously [27]. Beating cardiomyocytes were visible 12 days after differentiation and cardiomyocyte specific markers NKX2.5 and CTNT were detected by immunofluorescence staining (Fig. 3e).

### Clinical-grade hiPSCs are biologically safe

To evaluate whether our clinical-grade hiPSCs are biologically safe, we performed a series of tests according to the Guidelines for Human Somatic Cell Therapies and Quality Control of Cell-based Products. The results showed that the cells were negative for mycoplasma and free of serious pathogenic microorganisms such as HIV and HPV (Additional file 2). The endotoxin level of the hiPSC culture medium met the requirements defined in the *Pharmacopoeia of the People’s Republic of China*, 2010 edition, Volume III (Additional file 2). To detect other types of unknown pathogenic microorganisms, we injected the hiPSCs into the chorioallantoic and yolk sac of chicks. After 5 and 10 days of incubation separately, the chicks were still alive (Additional file 2), which indicated that the hiPSCs were not infected with pathogenic microorganisms.

## Discussion

Clinical-grade hiPSCs and GMP environments are essential in order to ensure the safety, effectiveness, traceability, and reproducibility of hiPSC-based clinical trials or therapies [16, 28]. To avoid the risk of cross-species infectious agent transfer, the usage of animal-source reagents in cell handling process is not recommended [29].

The hESCs used in the clinical trials approved by the US FDA as mentioned earlier were not derived under GMP conditions. Instead they were converted from research-grade lines which were derived and maintained in the presence of animal products [28]. It is unclear whether this protocol can be applied in hiPSCs. After all, it is far easier to obtain cell sources for hiPSCs than for hESCs. Recently, several groups reported that they have successfully derived clinical-grade or Xeno-free hiPSCs. However, the derivation of hiPSCs was not entirely Xeno-free [21–23] or under GMP conditions [20]. To our knowledge, our study is the first to report successful derivation of clinical-grade hiPSC lines completely under GMP conditions in Xeno-free reagents. These hiPSC lines may be a valuable source for future clinical trials or therapies.

Oncogenicity is a major concern for the application of hiPSCs in clinical settings. Nevertheless, an increasing number of studies in mouse models suggest that iPSCs are safe for transplantation [30]. For example, Tsuji et al. [30] have reported no tumor formation after the transplantation of iPSC-derived neural spheres into SCID mouse brain. The process of obtaining iPSCs is complex, however, involving the reconstruction of cellular epigenetic states. Assessment of genomic integrity and epigenetic variations of hiPSCs is therefore very important to ensure their safety. To date, the induction of genomic and epigenetic variations in iPSCs is still questionable. Hussein et al. [31] reported that there are more chromosomal copy number variations in iPSCs compared with embryonic stem cells and their parental cells, while Abyzov et al. [32] proved that reprogramming did not lead to copy number variations using single-cell sequencing. Interestingly, a few groups reported abnormal genomic imprinting and methylation in iPSCs [33, 34]. The quality of each iPSC line varies and thus it is important to define a series of criteria from the genomic and epigenetic level to evaluate the safety of the iPSCs before their clinical application. This requires joined efforts from scientists all over the world. The results of clinical trials in Japan will give us some clues.

Reprogramming efficiency is another important factor for future hiPSC-based therapies because parental cells from old patients which are difficult to be reprogrammed may be required. We compared the reprogramming efficiency of two Xeno-free mediums and found that the number of colonies yielded from X medium, defined in our laboratory, are three times that from pluriton™ reprogramming medium, suggesting that our X medium could be used as a high-efficiency clinical-grade reprogramming medium.

Envisaging the potential translation from hiPSCs to cell replacement therapies, Xeno-free and GMP conditions are essential for the differentiation of hiPSCs into functional cells. We demonstrated the feasibility of differentiating one clinical-grade hiPSC line to functional neurons and cardiomyocytes in Xeno-free mediums according to the monolayer differentiation methods. As for monolayer differentiation, each hPSC line has an optimal differentiation method, and hence further work will be focused on optimizing the monolayer differentiation method to obtain functional neurons, cardiomyocytes, and other cell types from the clinical-grade hiPSC lines.

The incidence of degenerative diseases such as Parkinson’s disease and Alzheimer’s disease which cannot be treated effectively by traditional medical methods is increasing worldwide. Theoretically, replacement of degenerated cells with normal functional cells in patients paves the way for new treatment strategies. hiPSCs can provide an unlimited number of cells for autologous or closely HLA-matched transplantation upon differentiation which significantly reduces the risk of host immune response in patients. Only by standardizing and normalizing all processes during stem cell-based therapies can we ensure therapy safety and obtain objective and reliable results. These processes involve acquisition, cultivation, characterization, and purification of stem cells, testing tumorigenicity and immunogenicity of stem cells, in vivo reinfusion, efficacy evaluation of stem cells, and so on. In the near future, stem cell-based therapies will create a landmark achievement in the field of regenerative medicine and provide the theoretical basis and technical supports for conquering particular “human ills” by following strict regulations.

## Conclusions

hiPSCs hold great promise in regenerative medicine. To ensure clinical safety and obtain reliable therapeutic results, standardized and normalized processes must be followed during hiPSC-based therapies. Herein, integration-free clinical-grade hiPSC lines were derived under GMP-controlled conditions and with Xeno-free reagent culture in line with the current guidance of international and national evaluation criteria, and proved to be biologically safe and capable of differentiating into functional neural cells and cardiomyocytes in Xeno-free culture media. These results provide that the clinical-grade hiPSC lines could be valuable sources for future hiPSC-based clinical trials or therapies, especially in neural and heart-related diseases.

## Additional files

Additional file 1: Table S1. Presenting primer sequences for RT-PCR. (DOC 37 kb) Additional file 2: Table S2. Presenting sterility and pathogen testing in the two clinical-grade hiPSC lines. (DOC 33 kb) Additional file 3: Figure S1. Showing characterization of another clinical-grade hiPSC line. A Morphology of one clinical-grade hiPSC grown on human foreskin fibroblasts feeder cells and in feeder-free conditions. Scale bar, 200 μm. B Clinical-grade hiPSCs express alkaline phosphatase. Scale bar, 200 μm. C Karyotyping results of one clinical-grade hiPSC line. D Immunofluorescence results of one clinical-grade hiPSC line. The hiPSCs express pluripotency markers OCT4, SOX2, SSEA-3, SSEA-4, and TRA-1-60 at the protein level and do not express differentiation marker SSEA-1. *SSEA* stage-specific embryonic antigen. Scale bar, 100 μm. E Morphology of EBs at day 8 derived from one clinical-grade hiPSC line. Scale bar, 200 μm. F Hematoxylin and eosin staining of teratoma derived from one clinical-grade hiPSC line. Scale bar, 100 μm. The teratomas contain tissues of all three germ layers. (TIFF 9074 kb)

## Abbreviations

BDNF: Brain-derived neurotrophic factor
bFGF: Basic fibroblast growth factor
DMEM: Dulbecco’s modified Eagle’s medium
EB: Embryoid body
EBV: Epstein–Barr virus
EDTA: Ethylenediamine tetraacetic acid
FDA: Food and Drug Administration
FITC: Fluorescein isothiocyanate
GDNF: Glial cell line-derived neurotrophic factor
GMP: Good manufacturing practice
HAV: Hepatitis A virus
HBV: Hepatitis B virus
HCMV: Human cytomegalovirus
HCV: Hepatitis C virus
hESC: Human embryonic stem cell
HFF: Human foreskin fibroblast
hiPSC: Human induced pluripotent stem cell
HIV: Human immunodeficiency virus
hLif: Leukemia inhibitory factor
hPSC: Human pluripotent stem cell
HPV: Human papilloma virus
HTLV-I: Human T-lymphotropic virus I
IACUC: Institutional Animal Care and Use Committee
JCV: John Cunningham virus
KOSR: Knockout serum replacement
NEAA: None essential amino acid
NIFDC: National Institutes for Food and Drug Control
NIH: National Institutes of Health
RPE: Retinal pigmented epithelial
SCID: Severe combined immunodeficiency
TGFβ3: Transforming growth factor type β3
