# Supplementary material for: Generation of clinical-grade human induced pluripotent stem cells in Xeno-free conditions
Source: Stem Cell Res Ther. 2015 Nov 12;6:223. doi: 10.1186/s13287-015-0206-y (PMC4643509; doi:10.1186/s13287-015-0206-y)
Supplement: Additional file 1: Table S1. — Presenting primer sequences for RT-PCR. (DOC 37 kb) [file 13287_2015_206_MOESM1_ESM.doc]

**Table S1: Primer seguences for RT-PCR**

| **Gene** | **Forward primer** | **Reverse primer** | **Amplicon size** |
| --- | --- | --- | --- |
| *Oct4* | CAAACTGAGGTGCCTGCCCTTC | ATTGAACTTCACCTTCCCTCCAACC | 188 |
| *Sox2* | GGGAAATGGGAGGGGTGCAAAAGAGG | TTGCGTGAGTGTGGATGGGATTGGTG | 161 |
| *Nanog* | AAGACAAGGTCCCGGTCAAG | CAGGCATCCCTGGTGGTAG | 296 |
| *E-cadherin* | TGGACCGAGAGAGTTTCCCT | ACGACGTTAGCCTCGTTCTC | 178 |
| *Rex1* | AAGGCAAGTCAAGCCAAGACC | TTCCAAAGAACATTCAAGGGAGC | 278 |
| *Gad1* | GCAACTGCTGGCACGACTGT | GCTTGTCTGGCTGGAAGAGGTAT | 295 |
| *Pax6* | GCCCGTCCATCTTTGCTTG | GCAGCCATCTTGCGTAGGTT | 251 |
| *Enolase* | CATCGGCATGGACGTAGCG | AGGCAGTTGCAGGACTTCTCGT | 297 |
| *Osteonectin* | ATGGTGCAGAGGAAACCGAAGA | GGCAAAGAAGTGGCAGGAAGAGT | 212 |
| *Nicastrin* | AAATGATGGGTTTGGTGTTTACTC | AAGAGCTGCATGGCACATAGTG | 231 |
| *Alpha-fetoprotein* | GAGCGGCTGACATTATTATCGG | ACCCTGAGCTTGGCACAGATC | 203 |
| *Exo-Sev* | GGATCACTAGGTGATATCGAGC | ACCAGACAAGAGTTTAAGAGATATGTATC | 181 |
| *Exo-Oct4* | ATGCACCGCTACGACGTGAGCGC | AATGTATCGAAGGTGCTCAA | 451 |
| *Exo-Sox2* | TTCCTGCATGCCAGAGGAGCCC | AATGTATCGAAGGTGCTCAA | 410 |
| *Exo-Klf4* | TAACTGACTAGCAGGCTTGTCG | TCCACATACAGTCCTGGATGATGATG | 532 |
| *Exo-c-Myc* | CCCGAAAGAGAAAGCGAACCAG | AATGTATCGAAGGTGCTCAA | 483 |
| *Gapdh* | AGGCATCCTCACCCTGAAGTA | CACACGCAGCTCATTGTAGA | 103 |
