# Supplementary material for: Generation of clinical-grade human induced pluripotent stem cells in Xeno-free conditions
Source: Stem Cell Res Ther. 2015 Nov 12;6:223. doi: 10.1186/s13287-015-0206-y (PMC4643509; doi:10.1186/s13287-015-0206-y)
Supplement: Additional file 2: Table S2. — Presenting sterility and pathogen testing in the two clinical-grade hiPSC lines. (DOC 33 kb) [file 13287_2015_206_MOESM2_ESM.doc]

**Table S2: Sterility and pathogen testing in the two clinical-grade hiPSC lines**

| **Sterility and pathogen** | **Results** |
| --- | --- |
| Mycoplasma | - |
| Human Papillomavirus (HPV) | - |
| Human Parvovirus B19 | - |
| Human Immuno Deficiency Virus I (HIV- I) | - |
| Human Immuno Deficiency Virus II (HIV- II) | - |
| John Cunningham virus (JCV) | - |
| Epstein-Barr virus (EBV) | - |
| Human Hepatitis C virus (HCV) | - |
| Human Hepatitus A Virus (HAV) | - |
| Human Cytomegalo Virus (HCMV) | - |
| Human T-lymphotropic Virus I (HTLV-I) | - |
| Human Hepatitis B virus (HBV) | - |
| Bovine virus | - |
| Porcine virus | - |
| Bovine serum albumin residuals | ＜ 5 ng/ml |
| Hemagglutination test of 9-11-day-old chick embryo allantoic fluid | Negative |
| Survival rateof 5-6-day-old chick embryo | ＞90% |
| Endotoxin level | ＜0.5 EU/ml |
