# Supplementary figures and images for: Generation of clinical-grade human induced pluripotent stem cells in Xeno-free conditions
Source: Stem Cell Res Ther. 2015 Nov 12;6:223. doi: 10.1186/s13287-015-0206-y (PMC4643509; doi:10.1186/s13287-015-0206-y)

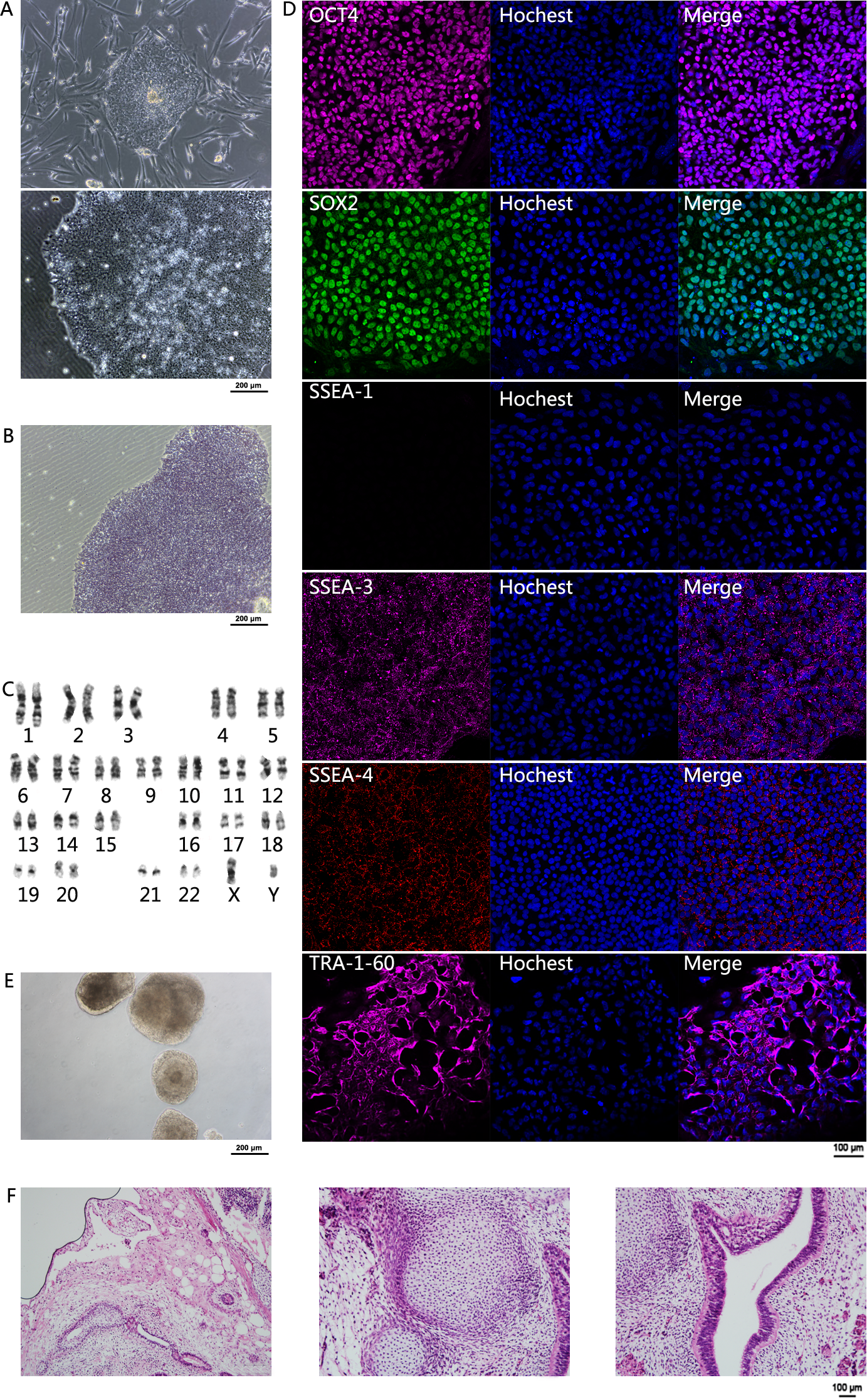

Supplement: Additional file 3: Figure S1. — Showing characterization of another clinical-grade hiPSC line. A Morphology of one clinical-grade hiPSC grown on human foreskin fibroblasts feeder cells and in feeder-free conditions. Scale bar, 200 μm. B Clinical-grade hiPSCs express alkaline phosphatase. Scale bar, 200 μm. C Karyotyping results of one clinical-grade hiPSC line. D Immunofluorescence results of one clinical-grade hiPSC line. The hiPSCs express pluripotency markers OCT4, SOX2, SSEA-3, SSEA-4, and TRA-1-60 at the protein level and do not express differentiation marker SSEA-1. SSEA stage-specific embryonic antigen. Scale bar, 100 μm. E Morphology of EBs at day 8 derived from one clinical-grade hiPSC line. Scale bar, 200 μm. F Hematoxylin and eosin staining of teratoma derived from one clinical-grade hiPSC line. Scale bar, 100 μm. The teratomas contain tissues of all three germ layers. (TIFF 9074 kb) [file 13287_2015_206_MOESM3_ESM.tiff]
